# Cervical Cancer Mortality in East-Central European Countries

**DOI:** 10.3390/ijerph17134639

**Published:** 2020-06-28

**Authors:** Cezary Wojtyla, Michal Ciebiera, Dariusz Kowalczyk, Grzegorz Panek

**Affiliations:** 1 Department of Oncologic Gynecology and Obstetrics, The Center of Postgraduate Medical Education, 00-416 Warsaw, Poland; gmpanek@wp.pl; 2 Second Department of Obstetrics and Gynecology, The Center of Postgraduate Medical Education, 01-809 Warsaw, Poland; michal.ciebiera@gmail.com; 3 Faculty of Health Sciences, State University of Applied Sciences, 62-800 Kalisz, Poland; dariusz.w.kowalczyk@wco.pl

**Keywords:** cervix uteri, epidemiology, screening, mortality, time trends, east-central Europe

## Abstract

Changes that took place in Europe in the early 1990s had an impact on health-associated issues. They were an impulse for the changes in healthcare systems and, consequently, also for the changes in cancer control programmes. Those changes also had an effect on mortality rates due to cervical cancer (CC). Therefore, the aim of this study is to analyse CC mortality trends in east-central Europe after 1990. Data on deaths due to CC were retrieved from the WHO Mortality Database. Trends in east-central European countries between 1990 and 2017 were assessed using Joinpoint Regression Program software. CC mortality decreased in the majority of analysed countries. However, an increase was observed in Latvia and Bulgaria. Despite decreasing mortality in the majority of the analysed countries, significant differences were observed. In order to improve the epidemiological situation, effective early detection programmes for cervical cancer ought to be rearranged and based not only on pap smears but also on molecular methods, as well as on introducing widespread programmes of vaccination against HPV.

## 1. Introduction

Cervical cancer (CC) is the ninth most frequently diagnosed neoplastic disease in European women and the second most common tumour occurring in women aged 15–44 years [1]. As the cause of death from cancer, it occupies the eleventh place in Europe and holds second place in younger women [1]. However, significant differences occur between European countries in terms of the incidence and mortality of cervical cancer. According to the Catalan Institute of Oncology and the International Agency for Research on Cancer, incidence rates in 2018 were 16.0/100,000 for women in Eastern Europe, and 6.8/100,000 for women in Western Europe, and mortality rates were 6.1/100,000 and 2.1/100,000, respectively [1]. 

The east-central European region has 80 million female inhabitants aged over 15 years, who are the future risk group for cervical cancer [1]. The region may be defined by the following countries: Albania, Bosnia and Herzegovina, Belarus, Bulgaria, Croatia, the Czech Republic, Estonia, Hungary, Latvia, Lithuania, Macedonia, Montenegro, Moldova, Poland, Romania, Serbia, Slovakia, Slovenia, and Ukraine [1]. This regional division is not only geographically important, but it also encompasses the countries belonging to the former Eastern Bloc, a system that had governed health policy for several decades very differently from Western Europe. It has undoubtedly contributed to the inequalities regarding the epidemiology of cervical cancer in Europe. Therefore, the aim of this study is to analyse CC mortality trends in east-central Europe after 1990.

## 2. Materials and Methods

CC mortality trends were investigated in this study for all the countries of central and eastern Europe (CEE), as aforementioned, except some parts of Albania, Bosnia and Herzegovina, and Montenegro due to incomplete data. A commonly adopted term used in comparative analyses is EU10, referring to the ten east-central countries which joined the European Union (EU) in 2004 and 2007 (i.e., Bulgaria, the Czech Republic, Estonia, Hungary, Latvia, Lithuania, Poland, Romania, Slovakia, and Slovenia), whereas the term EU15 refers to the EU Member States prior to 2004 (i.e., Austria, Belgium, Denmark, Finland, France, Greece, Spain, the Netherlands, Ireland, Luxembourg, Germany, Portugal, Sweden, Great Britain, and Italy).

Data on CC mortality for every analysed country were obtained from the World Health Organization Mortality Database between 1990 (or in the first year after 1990 for which such data were available) and 2017 (or in the last available year before 2017). This database provides information concerning the number of deaths by causes defined in the International Classification of Diseases (ICD) according to gender, age and calendar year. Our study analysed the standardised mortality rates due to CC in European countries according to ICD-9 diagnosis code 180 and ICD-10 code C53. Cases encoded as cervical cancer, cancer of the corpus uteri and unspecified uterine cancer were analysed together for the group of women aged 15–44. It stems from the fact of a discrepancy, which was observed when coding the causes of deaths due to uterine cancers and the fact that the majority of deaths due to uterine cancers were caused by CC among women in this age group [2]. The standard world population was used to calculate standardised mortality rates. No extrapolation was made for any missing data.

The analysis of the total cervical cancer mortality rates in CEE countries between 1990 and 2015 was performed using the Joinpoint Regression Program ver. 4.5.0.1. This software was used to test whether time trend factors or their changes within the studied time interval were statistically significant (*p* < 0.05). The maximum number of joinpoints was set to 3. In addition, we calculated the average annual percentage change (APC) between 1990 and 2017, with the number of joinpoints set to 0.

## 3. Results

The mortality rates of CC are shown in Table 1 for women of all ages (0+) in CEE from the beginning of the observation period (either in 1990 or in the first year after 1990 for which the data were available) till the end of the observation in 2017 (or in the last available year before 2017). The rates of change between 1990 and 2017 are also expressed as APCs. In 1990, the highest rates were noted in women from Romania, Poland, Hungary and Serbia. They ranged from 7.0/100,000 women in Serbia and Hungary to 10.1/100,000 women in Romania. The mortality rate for EU10 was 9.6/100,000 women. At that time, EU15 presented the mortality rate at the level of 3.5/100,000 women. Macedonia and Croatia were the only countries with the rates lower than the EU15 mean. In 2017, the rates were lower for the majority of CEE countries compared to 1990. We also observed a decrease in APC for most of them. However, it increased in Latvia and Bulgaria. The largest decreases in the mortality rate expressed as APC occurred in Poland, the Czech Republic, and Slovenia and were twice as high as the APC for EU10. In 2017, the highest rates were noted in Romania, Serbia, Moldova, Lithuania, and Latvia, whilst the lowest ones in Slovenia, Croatia, and Macedonia. CC mortality is still lower in Western Europe, and its rate of decrease is higher compared to the CEE countries.

**Figure 1:**
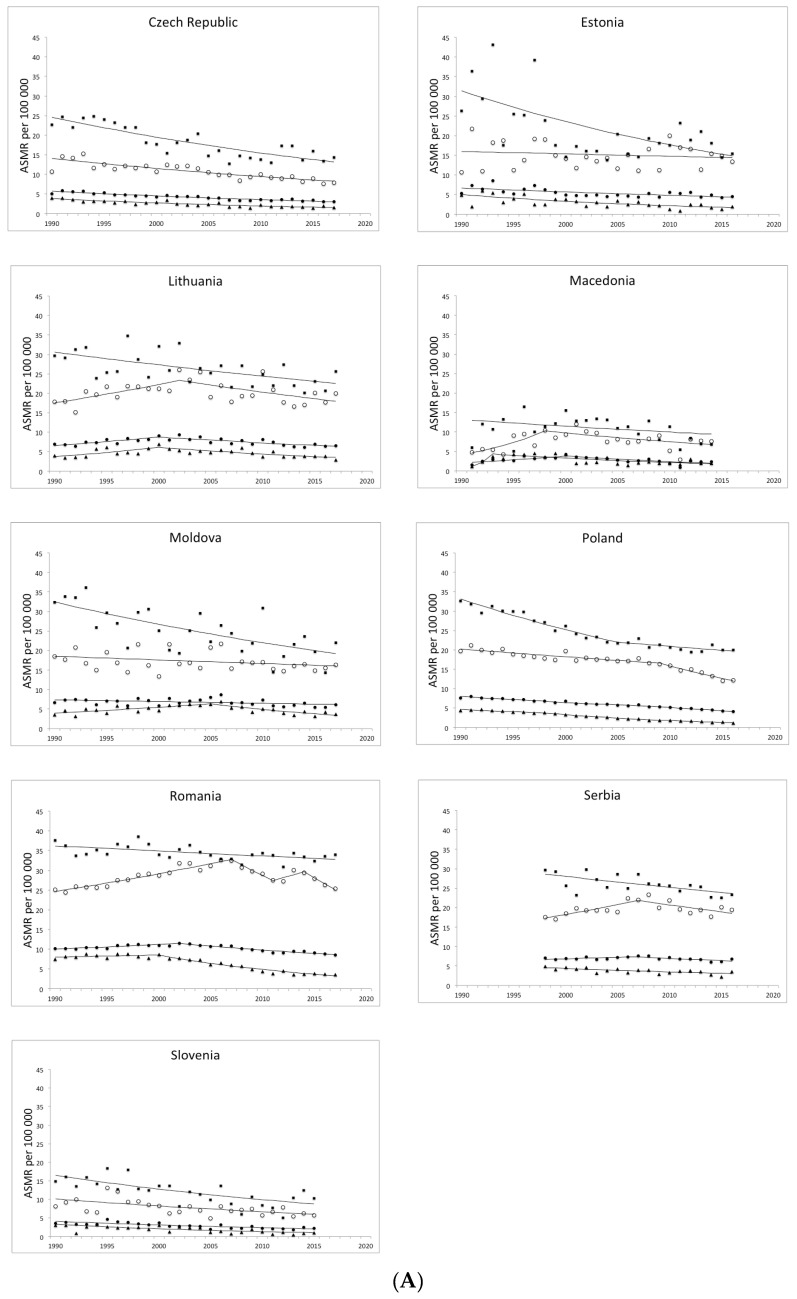

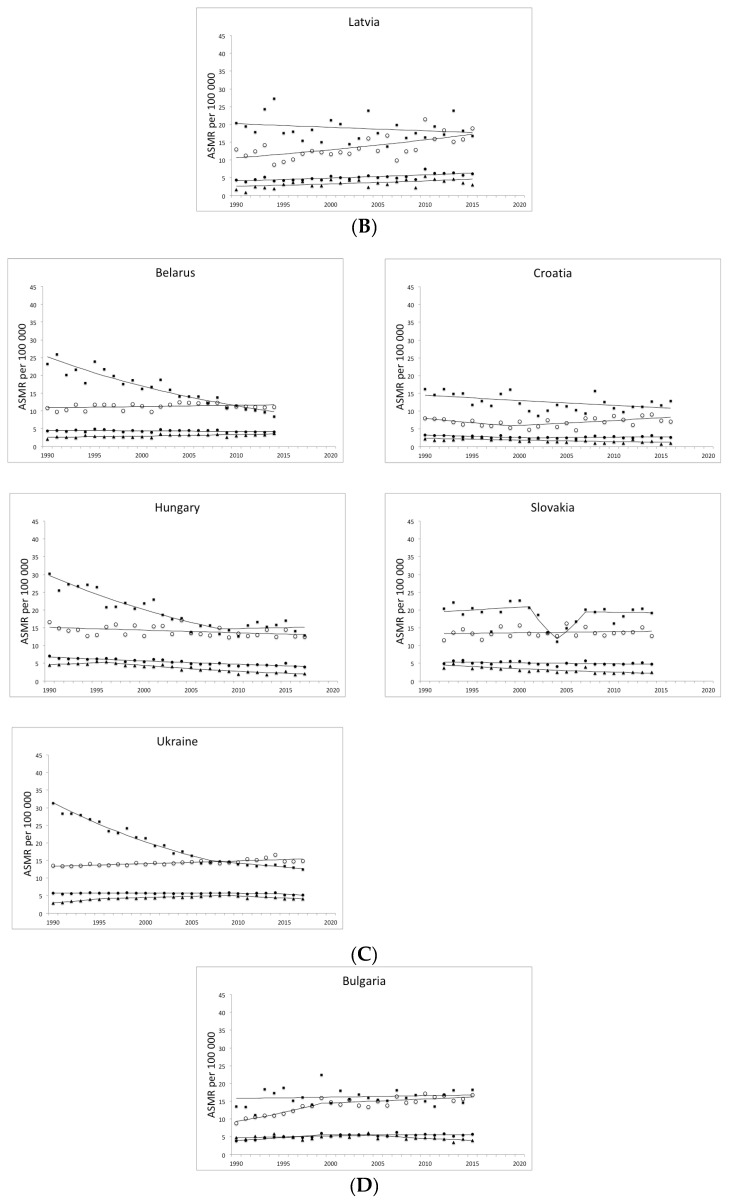
Joinpoint analysis of trends in age-standardised mortality rates due to cervical cancer in women of all ages (a solid circle), 15–44 (a triangle), 45–64 (an empty circle) and over 65 (a square) years old during the period 1990–2017. ASMR—age-standardised mortality rates. (**A**)—first pattern; (**B**)—second pattern; (**C**)—third pattern; (**D**)—fourth pattern.

The joinpoint regression analysis results on CC mortality trends for CEE countries are shown in Table 2 and Figure 1. The countries studied may be divided into several categories according to cervical cancer mortality trends. The first one (Figure 1A) is observed in those countries where mortality in women of all ages decreased (i.e., the Czech Republic, Estonia, Slovenia, Lithuania, Moldova, Macedonia, Serbia, Poland and Romania). In the Czech Republic, Estonia, Poland and Slovenia, such decreases had been noted since the beginning of the observation. In Lithuania, Moldova, Macedonia, Serbia, and Romania, the trends changed during the observation. The second group comprised Latvia (Figure 1B), where cervical cancer mortality increased in all age groups. However, mortality in the oldest women slightly decreased, but the change was insignificant. The remaining countries demonstrated disproportionate mortality trends for various age groups of women. Nevertheless, they were defined according to the category of trend change observed in all women (0+). In Hungary, Belarus, Slovakia, Ukraine and Croatia, we observed a decrease in mortality in this age group (Figure 1C). In Hungary, the oldest group of women (65+) was the only one with an increasing trend. However, it was insignificant. In Belarus, we observed an increasing trend in the youngest (15–44 years old) and middle-aged (45–54 years old) groups of women. The trend in the oldest group was decreasing. In Slovakia, an insignificant increasing trend was observed only in the middle-aged group. The situation was the same in Ukraine, but the increasing trend in middle-aged women was statistically significant. We also included Croatia into this group, but an insignificant change of trend in the 0+ group was observed. Moreover, an increasing trend of mortality had been present in the middle-aged group since 1999. In the remaining groups, the trend was decreasing. Finally, Bulgaria had been characterised by increasing mortality in the 0+ group of women until 1999, when it reached a plateau (Figure 1D). The only group with a decreasing trend was the youngest one.

## 4. Discussion

Our study began in 1990, when the Iron Curtain in Europe collapsed, which marked the beginning of numerous political, economic, social, and public health-related changes. However, those changes were not rapid. For many years, east-central European countries have been struggling with many problems left over from the previous political system. Some changes in healthcare made in the 1980s [3] in the Eastern Bloc had persisted. Furthermore, the importance of clinical medicine had begun to be emphasised regarding public health. It survived the collapse of the Iron Curtain in many central and eastern European countries [4,5,6]. However, at that time, important population-based and organised early-detection programmes for cervical cancer were not undertaken, as was the case in numerous western European countries [7]. Therefore, cervical cancer mortality rates remained high in CEE countries at the beginning of the 1990s, contributing to a significant disproportion between western and CEE countries [8]. In 1993, the first edition of the European Guidelines for cervical cancer screening was issued, which set out the basic principles for detecting cervical cancer [9]. The health ministers of EU countries signed the European Council Recommendation on Cancer Screening in 2003, which included the organisation of CC screening [10]. It was then backed up with the second edition issued in 2008, with recommendations for decision-makers on how a well-functioning screening programme should be organised [11]. In 2004, 2007 and 2013, numerous CEE countries joined the EU and were obliged to resolve the problem of high CC mortality. As a result, many countries launched programmes for the early detection of this disease [12]. However, their effectiveness depended largely on the coverage of the target population [13]. Moreover, organised screening, as opposed to opportunistic screening, is more cost-effective, because it only examines a defined target population at appropriate time intervals. It also provides quality assurance at all levels and evaluates programme effectiveness over time [11,14].

Not all CEE countries have managed or decided to introduce such screening programmes for reducing CC mortality, despite their proven benefits. Those countries which did found differences in how they were organised, their target populations, screening methods (e.g., conventional cytology or liquid-based cytology) and whether HPV was investigated in the programme [15]. Studies by Elfström et al. [15] and by Poljaket et al. [12] investigated the functioning of screening programmes in selected European countries and found that opportunistic programmes were still adopted in Bosnia and Herzegovina, Macedonia, Montenegro, Serbia, Bulgaria, and Slovakia. The programme introduced in the Czech Republic was non-population-based. The screening coverage of the east-central target population of Europe was also lower than in western Europe and exceeded 60% only in Slovenia [15]. Only in the Czech Republic, Latvia, and Slovenia, all quality indicators of the screening register were tracked (i.e., comparability, completeness, validity and timelines). An organised screening programme is one where monitoring and evaluation are audited. Unfortunately, it has not been undertaken in the majority of CEE countries [15].

The epidemiological situation in CEE countries has changed considerably over the last decades. A study by Arbyn et al. [16] on the Baltic countries indicated that CC mortality had increased in Latvia and Lithuania between the early 1980s and 2004 in almost all age groups, but it was stable in Estonia. A similar upward trend (APCs) had been observed in Bulgaria and Romania since 1980. Between 1989 and 2004, an upward trend still continued in Bulgaria, but it became insignificant. A slight downward trend had been observed in the mortality of Bulgarian and Romanian women aged over 55 years from 1985 onwards, whilst those aged 20–59 overall showed a concomitant increase, with the exception of the 20–29 year age subdivision. 

As regards some other countries of the tested region, CC mortality for all ages decreased in Bosnia and Herzegovina, Croatia, the Czech Republic, Hungary, Poland, Slovakia, and Slovenia [17,18,19]. However, the trend increased in Belarus, Moldova (until the first part of the 2000s), and Ukraine. In Albania, it remained relatively stable [17,18]. 

CC mortality trends were investigated in another study by Arbyn et al. [17]. They analysed selected age groups of women, dating back to between the first part of the 1950s and 2005. The groups were found to vary significantly over the decades, according to the country and age group. A long-standing downward trend in all age groups was observed in the Czech Republic, Poland, and Hungary. The present study demonstrates that this trend continued on in the Czech Republic and Poland. Furthermore, other countries have joined this decreasing trend (Estonia, Slovenia, Lithuania, Moldova, Macedonia, Serbia and Romania). Hungary was excluded because trends in women aged above 65 were insignificantly changed. The aforementioned study also revealed decreasing mortality in young Croatian women, below 44 years, and in those aged 60–64 years until the 1980s, but an upward trend in all the other age groups [17]. Our observations confirm that the downward trend in young adult women continued but was also apparent in those aged over 65 years. In our study, women were divided into slightly different age groups. Nevertheless, we also found upward trends in other age groups. The study by Arbyn et al. also showed increased mortality rates in Macedonia and Slovakia in almost all age groups of women between 1990 and 2000, except for women aged 30–34 and 70–74 years, where the mortality was decreased [17]. Our study reveals that the changed trends in Macedonia qualifies this country for the group with decreasing mortality trends in all age groups. In Slovakia, the trend was upward in women aged over 44 years, whilst it was downward for younger women. The study by Arbyn et al. also investigated mortality trends in Bosnia and Herzegovina (1985–1990), as well as Serbia, Montenegro (1995–2000), and Albania. Women’s mortality increased in the group under 44 years in Bosnia and Herzegovina, Serbia and Montenegro, but decreased in all other age groups. In Albania, the mortality decreased in the groups under 34 and 80–84 years between 1985 and 2000, whereas it increased at ages 40–44 years, and was unstably irregular for the other age groups [17]. In Serbia, we observed decreasing trends for all groups of women. The study by Arbyn et al. showed increased mortality in Belarus (between 1980–2000) in women under 54 years, but a downward trend in all the other age groups for the last few years of the study period [17]. We also found declining mortality trends over our entire observation period in women over 65 years of age, as well as the all-women group (0+). Increases were seen for women aged up to 65 years. In Slovenia and Moldova, decreases were observed by Arbyn et al. in women under 54 and in those aged 80–84 years, whereas an upward trend was found for all the other age groups [17]. In our study, both those countries qualify as presenting falling mortality trends for all age groups of women. Finally, the study by Arbyn et al. demonstrated increased mortality rates in Ukraine in almost all age groups of women, except for those aged 70–84 years [17]. The situation in Ukraine has changed in recent years. Our observations indicate a recent drop in the mortality trend for the youngest group of women aged 15–44 years and also a downward trend in the oldest group of women in our study (65+ years) and for the 0+ group. Nonetheless, the upward trend is still observed in women aged 45–64 years.

When investigating trends in CC mortality dating back several decades, several problems are encountered, such as inconsistencies in classifying reproductive organ tumors and the fact that the International Classification of Diseases has been modified several times over recent years. Death certificates do not precisely distinguish between cervical and corpus uteri cancers since they were mostly classified as being unspecified uterine cancers. Upon reviewing the data of specific countries over recent decades, it is noticeable that up to 93% of all uterine cancers were diagnosed in such a way, for example, in Spain in 1960 [8]. In contrast, the UK classified unspecified uterine cancers only in 6% of cases at that time. However, in 1995, such diagnoses in Spain were made in 32% of cases, whereas in the UK, the rate was 21% of cases [8]. Mortality due to cervical cancer is, therefore, affected by misclassification bias. Some studies, for example, by Levi at al. and Wojtyla et al., have tried to minimise the problem by investigating the mortality only in young women, in whom cervical tumors constitute the overwhelming majority of all uterine tumors [2,8]. To our knowledge, this paper is the first one to illustrate the mortality of women due to CC in all age groups of women living in the region of central and eastern Europe using a separate method of classifying deaths among young adult women. In this way, it allows the minimisation of the classification bias and more reliable comparison of the results between this group and other groups of women. 

Our study has several limitations. A change of the International Classifications of Diseases was made during the period covered by our analysis. Individual countries introduced the new classification at different times. In addition, uterine cancers may be classified as cancer of the corpus uteri, cervix or unspecified parts of the uterus. Both of the above factors make the epidemiological analysis of cervical cancer prone to classification bias. It is irrelevant in case of changes in the last two International Classifications of Diseases because all three abovementioned codes have their equivalents in individual classifications. However, it affects the loss of some cervical cancers classified as unspecified uterine cancer. The bias can only be minimised for younger women by assuming that uterine cancers in this age group (under 45 years old) are cervical cancers. As mentioned before, the vast majority of uterine cancers in this age group are cervical cancers [2,8]. However, this methodology cannot be applied to other groups of women. Another limitation stems from the comparison of countries with different populations. Obviously, mortality in our analysis is presented in the form of standardised mortality rates using the world standard population. Nevertheless, the number of deaths in some countries is low and small changes in the number of deaths may exert a significant impact on observed trends.

Despite a great deal of effort devoted to reducing and achieving low rates of CC mortality, as observed in most western and northern European countries, it is still high in most CEE countries. This study confirms previous observations on the inequalities in female mortality from cervical cancer in Europe. Nevertheless, the observations are based on the latest data. However, epidemiological studies of this type are used to shape the current health policy of individual countries and individual health areas. The observations described in our paper should encourage decision-makers to increase their efforts to improve the situation, especially where there are available tools to achieve it. Poorly functioning screening programmes are the main reason responsible. However, the case of Slovenia is exceptional. It is the only country in the region with a well-functioning and effective screening programme. It included 82.1% of women (data from 2004–2008) and led to a 40% fall in the cervical cancer incidence rate during 2003–2009 [20]. Great hopes are currently pinned upon protective HPV vaccines. The vaccines, coupled with fully functioning and organised early-detection programmes for CC, may result in this disease being no longer regarded as a public health issue in the world. Hall MT et al. [21]. performed a simulation of what the expected CC incidence and mortality rates in Australia could be if high-coverage protective HPV vaccination programmes were introduced. They found that in a short time, CC could be eliminated from the population. The time for eradication is much shorter than that achieved by screening alone, where the obtained levels of incidence and mortality are significantly lower. Therefore, it appears that only the simultaneous introduction of appropriate screening programmes coupled with high-coverage protective vaccinations may lead to the situation when the level of CC mortality in CEE countries approximates the one observed in Western Europe at the moment. 

Nevertheless, there is one more issue that may have an enormous impact on perpetuating the positive mortality trend generated by the above factors. It is associated with the organisation of appropriate educational programmes concerning cervical cancer prevention for young women. Those women are mostly included in the current cervical cancer prevention programme in a given country because it shapes their awareness of cervical cancer prevention for their entire future life. Sudden changes in the organisation of screening will have a lower impact on women from older age groups because their attitude towards cervical cancer prevention has already been shaped. Young women may not be fully educated and aware of the threat yet, because they have not developed the habit of undergoing routine screening. New diagnostic methods and changes in screening programmes will be ineffective if women are unaware of the threat and the possibilities of its prevention.

## 5. Conclusions

Although CC mortality has decreased in the majority of CEE countries over recent decades, there are still disproportions between particular countries, as well as between age groups of women within individual countries from this region of Europe. These countries present significantly higher mortality rates than western Europe. In order to improve the epidemiological situation for the coming years, early detection programmes ought to be reorganised and based not only on pap smears but also on molecular methods, together with widespread programmes of vaccination against HPV. 

## Figures and Tables

**Table 1:** Cervical cancer data availability, mortality rates and annual percentage change (APC) in all age groups of women (0+).

Country	Years	Cervical Cancer Mortality Rates (per 100,000 Population)		
		1990 ^a^	2017 ^b^	APC 1990 ^a^ and 2017 ^b^
Latvia	1990–2015	4.4	6.1	1.7
Bulgaria	1990–2015	3.9	5.7	1.1
Ukraine	1990–2017	5.7	5.2	−0.2
Serbia	1998–2016	7.0	6.7	−0.4
Lithuania	1900–2017	6.9	6.6	−0.4
Belarus	1990–2014	4.4	4.1	−0.4
Croatia	1990–2016	3.4	2.6	−0.5
Slovakia	1992–2014	5.0	4.8	−0.5
Romania	1990–2017	10.1	8.5	−0.6
Moldova	1990–2017	6.7	6.1	−0.7
Macedonia	1991–2014	1.5	2.4	−1.2
Estonia	1990–2016	5.3	4.5	−1.6
Hungary	1990–2017	7.0	4.0	−1.8
the Czech Republic	1990–2017	5.0	3.1	−2.3
Poland	1990–2016	7.7	4.1	−2.3
Slovenia	1990–2015	3.6	2.2	−2.7
UE10	1990–2014	9.6	7.3	−1.2
UE15	1990–2015	3.5	1.9	−2.5

^a^ Or the first available year after 1990; ^b^ or the last available year before 2017.

**Table 2:** Joinpoint analysis for cervical cancer mortality of women at all ages (0+), 15–44, 45–64 and over 65 (65+) years old, by country.

	0+	15–44	45–54	65+
**Belarus**				
APC 1 (Period 1)	−0.4 * (1990–2014)	1.3 * (1990–2014)	0.3 (1990–2014)	−3.9 * (1990–2014)
**Bulgaria**				
APC 1 (Period 1)	3.8 * (1990–1999)	1.0 (1990–2004)	5.0 * (1990–1999)	0.2 (1990–2015)
APC 2 (Period 2)	0.0 (1999–2015)	−2.8 * (2004–2015)	0.7 * (1999–2015)	
**Croatia**				
APC 1 (Period 1)	−2.4 * (1990–2001)	−2.5 (1990–2016)	−3.3 (1990–1999)	−1.1 * (1990–2016)
APC 2 (Period 2)	0.8 (2001–2016)		2.1 * (1999–2016)	
**the Czech Republic**				
APC 1 (Period 1)	−2.3 * (1990–2017)	−3.3 * (1990–2017)	−2.9 * (1990–2017)	−1.8 (1990–2017)
**Estonia**				
APC 1 (Period 1)	−1.6 * (1990–2016)	−4.1 * (1990–2016)	−0.4 (1990–2016)	−2.8 * (1990–2016)
**Hungary**				
APC 1 (Period 1)	−1.8 * (1990–2017)	2.2 (1990–1996)	−0.5 * (1990–2017)	−3.8 * (1990–2008)
APC 2 (Period 2)		−4.5 * (1996–2017)		0.4 (2008–2017)
**Latvia**				
APC 1 (Period 1)	1.7 * (1990–2015)	2.4 * (1990–2015)	2.0 * (1990–2015)	−0.5 (1990–2015)
**Lithuania**				
APC 1 (Period 1)	2.9 * (1990–2000)	5.1 * (1990–2000)	2.4 * (1990–2002)	−1.1 (1990–2017)
APC 2 (Period 2)	−1.9 * (2000–2017)	−3.2 * (2000–2017)	−1.7 * (2002–2017)	
**Macedonia**				
APC 1 (Period 1)	6.2 * (1991–2000)	95.0 (1990–1993)	11.8 * (1991–1998)	−1.4 (1991–2014)
APC 2 (Period 2)	−4.7 * (2000–2014)	−4.0 (1993–2014)	−2.6 * (1998–2014)	
**Moldova**				
APC 1 (Period 1)	−0.7 * (1990–2017)	3.1 * (1990–2005)	−0.5 (1990–2017)	−1.9 * (1990–2017)
APC 2 (Period 2)		−5.0 * (2005–2017)		
**Poland**				
APC 1 (Period 1)	−2.1 * (1990–2009)	−2.6 * (1990–1998)	−1.0 * (1990–2009)	−2.7 * (1990–2005)
APC 2 (Period 2)	−3.5 * (2009–2016)	−6.1 * (1998–2016)	−4.4 * (2009–2016)	−1.0 * (2005–2016)
**Romania**				
APC 1 (Period 1)	1.1 * (1990–2002)	0.6 (1990–2000)	1.7 * (1990–2007)	−0.4 * (1990–2017)
APC 2 (Period 2)	−1.9 * (2002–2017)	−5.5 * (2000–2017)	−4.4 * (2007–2011)	
APC 3 (Period 3)			2.7 (2011–2014)	
APC 4 (Period 4)			−5.4 * (2014–2017)	
**Serbia**				
APC 1 (Period 1)	1.0 (1998–2007)	−2.2 * (1998–2016)	2.6 * (1998–2007)	−1.1 * (1998–2016)
APC 2 (Period 2)	−1.8 (2007–2016)		−1.8 * (2007–2016)	
**Slovakia**				
APC 1 (Period 1)	−0.5 * (1992–2014)	−3.2* (1992–2014)	0.2 (1992–2014)	0.7 (1992–2001)
APC 1 (Period 2)				−17.3 (2001–2004)
APC 3 (Period 3)				18.1 (2004–2007)
APC 4 (Period 4)				−0.2 (2007–2014)
**Slovenia**				
APC 1 (Period 1)	−2.7 * (1990–2015)	−4.5 * (1990–2015)	−2.0 * (1990–2015)	−2.5 * (1990–2015)
**Ukraine**				
APC 1 (Period 1)	0.0 (1990–2014)	7.1 * (1990–1995)	0.5* (1990–2017)	−4.3 * (1990–2007)
APC 2 (Period 2)	−3.3 * (2014–2017)	1.5 * (1995–2009)		−1.6 * (2007–2017)
APC 3 (Period 3)		−2.7 * (2009–2017)		

* statistically significant, *p* < 0.05.
